# Humanized Mouse Models for the Study of Periodontitis: An Opportunity to Elucidate Unresolved Aspects of Its Immunopathogenesis and Analyze New Immunotherapeutic Strategies

**DOI:** 10.3389/fimmu.2021.663328

**Published:** 2021-06-17

**Authors:** Carolina Rojas, Michelle P. García, Alan F. Polanco, Luis González-Osuna, Alfredo Sierra-Cristancho, Samanta Melgar-Rodríguez, Emilio A. Cafferata, Rolando Vernal

**Affiliations:** ^1^Periodontal Biology Laboratory, Faculty of Dentistry, Universidad de Chile, Santiago, Chile; ^2^Faculty of Dentistry, Universidad Andres Bello, Santiago, Chile; ^3^Department of Conservative Dentistry, Faculty of Dentistry, Universidad de Chile, Santiago, Chile; ^4^Department of Periodontology, School of Dentistry, Universidad Científica del Sur, Lima, Perú

**Keywords:** periodontitis, animal model, humanized mice, immunopathogenesis, immunotherapy

## Abstract

Periodontitis is an oral inflammatory disease in which the polymicrobial synergy and dysbiosis of the subgingival microbiota trigger a deregulated host immune response, that leads to the breakdown of tooth-supporting tissues and finally tooth loss. Periodontitis is characterized by the increased pathogenic activity of T helper type 17 (Th17) lymphocytes and defective immunoregulation mediated by phenotypically unstable T regulatory (Treg), lymphocytes, incapable of resolving the bone-resorbing inflammatory milieu. In this context, the complexity of the immune response orchestrated against the microbial challenge during periodontitis has made the study of its pathogenesis and therapy difficult and limited. Indeed, the ethical limitations that accompany human studies can lead to an insufficient etiopathogenic understanding of the disease and consequently, biased treatment decision-making. Alternatively, animal models allow us to manage these difficulties and give us the opportunity to partially emulate the etiopathogenesis of periodontitis by inoculating periodontopathogenic bacteria or by placing bacteria-accumulating ligatures around the teeth; however, these models still have limited translational application in humans. Accordingly, humanized animal models are able to emulate human-like complex networks of immune responses by engrafting human cells or tissues into specific strains of immunodeficient mice. Their characteristics enable a viable time window for the study of the establishment of a specific human immune response pattern in an *in vivo* setting and could be exploited for a wider study of the etiopathogenesis and/or treatment of periodontitis. For instance, the antigen-specific response of human dendritic cells against the periodontopathogen *Porphyromonas gingivalis* favoring the Th17/Treg response has already been tested in humanized mice models. Hypothetically, the proper emulation of periodontal dysbiosis in a humanized animal could give insights into the subtle molecular characteristics of a human-like local and systemic immune response during periodontitis and support the design of novel immunotherapeutic strategies. Therefore, the aims of this review are: To elucidate how the microbiota-elicited immunopathogenesis of periodontitis can be potentially emulated in humanized mouse models, to highlight their advantages and limitations in comparison with the already available experimental periodontitis non-humanized animal models, and to discuss the potential translational application of using these models for periodontitis immunotherapeutics.

## Introduction

The oral mucosa is a place of immense antigenic diversity that demands a tightly balanced immune surveillance, capable of maintaining the balance between host and microbial interactions. Indeed, a plethora of signals, including food antigens, airborne particles, commensal microbiota, and ongoing damage from mastication, finely tune the oral mucosal barrier immunity (1, 2). Moreover, the existence of an incredibly thin and highly-permeable epithelium located at the bottom of the gingival crevice, composed of 3-5 layers of cells and harboring diverse microbial communities between the tooth and the gingiva, allows the continuous transmigration of immune cells against the microbial challenge, thus making this scenario particularly challenging for a balanced immune response (1, 3).

## Etiopathogenesis of Periodontitis: Microbiota and Host Interactions

Certainly, constant environmental stimuli given by oral anatomical features and their functions, as well as the salivary flow and composition, directly influence the tooth-adherent microbiota. Hence, the orchestrated stability of at least 700 different taxa colonizing these distinct oral meta-niches has been a permanent matter of attention for dentists (4). In a healthy periodontium, the symbiosis between the resident eubiotic microorganisms and the host’s immune response commands this equilibrium. However, ill-defined factors, such as plaque accumulation, diet, stress, smoking, genetic predisposition, chronic inflammation, among others, contribute to a disequilibrium within bacterial communities, favoring the emergence of highly virulent bacteria, including *Porphyromonas gingivalis*, *Aggregatibacter actinomycetemcomitans*, and *Fusobacterium nucleatum*, as well as the reduction of health-compatible commensal bacteria.

For instance, *P. gingivalis* pathogenicity relies on its capability of expressing a variety of virulence factors, such as lipopolysaccharide, extracellular capsule, gingipains, and fimbriae, which, despite its low relative abundance, can both over-activate and subvert the immune response in mice or human periodontium (5, 6). Furthermore, different strains of this keystone pathogen have been detected in active periodontal lesions of teeth with poor prognosis and have demonstrated the capacity of eliciting a differential osteo-destructive immune response (7–9). Otherwise, the pathobiont *A. actinomycetemcomitans*, associated with more severe forms of periodontitis, is also armed with a variety of virulence factors such as lipopolysaccharide, leukotoxin, and fimbriae, which induce leukocyte lysis, favor periodontal colonization and provoke a dysregulated immune response during periodontal inflammation (10–12). In this context, the polymicrobial synergy of a dysbiotic bacterial consortium, frequently including the interplay between *P. gingivalis*, *A. actinomycetemcomitans*, and/or *F. nucleatum*, leads to a cycle of pathogenic inflammation, which perpetuates a nutrient-rich environment that promotes their expansion and deeper invasion of periodontal tissues; thus, provoking inflammatory alveolar bone loss in a susceptible host (13, 14).

Apart from other mucosal barriers entirely dependent on microbial commensals, gingival immune homeostasis is distinctly influenced by physiological damage during mastication. In fact, chewing elicits the production of interleukin (IL)-6 by fibroblasts, which promotes oral barrier protection *via* T-helper type 17 (Th17)-mediated immunity (15). Indeed, Th17 lymphocytes are key players in the maintenance of oral integrity by actively recruiting neutrophils to the teeth/mucosa interface and controlling opportunistic fungal infections (16); however, they are also key drivers of osteolytic inflammation during periodontitis. Thus, the amplification and dysregulation of Th17 lymphocyte activity mediated by Th17-related cytokines, including IL-6, IL-17A, IL-21, IL-23, and the osteolytic factor termed receptor activator of nuclear factor κB ligand (RANKL), lead to the breakdown of soft tooth-supporting tissues and alveolar bone resorption in susceptible individuals (16–18).

Contrarily, the maintenance of tolerance, prevention of autoimmunity, and inhibition of chronic inflammation required in the healthy periodontium has been attributed to T regulatory (Treg) lymphocyte activity (19–21). Treg lymphocytes are mainly characterized by their sustained surface expression of the IL-2 receptor α chain, termed CD25, and their signature transcription factor forkhead box P3 (Foxp3), and by having an armament of molecular strategies for, as its name implies, regulate both the innate and adaptive immune response (22). Treg lymphocytes control, at least in part, periodontal inflammation *via* the production of immunoregulatory cytokines, such as transforming growth factor (TGF)-β1, IL-10, and IL-35, which inhibit the expansion and activity of effector T lymphocytes such as Th1 and Th17 cells (21, 23). Periodontitis-affected tissues show enrichment in Treg lymphocyte activity demonstrated by the increased expression of their associated immune-regulatory/suppressive molecules like cytotoxic T-lymphocyte antigen 4 (CTLA-4), glucocorticoid-induced TNF-related protein (GITR) and Foxp3 (24). On the other hand, systemic ablation of Treg lymphocytes, by inoculating anti-GITR, increases the periodontal levels of tumor necrosis factor (TNF)-α, RANKL, and alveolar bone loss (25). However, for these immune-regulatory properties to be effective, the maintenance of their regulatory phenotype, mediated by the expression of Foxp3, is mandatory (20, 26). During periodontitis, Treg lymphocytes show a reduced Foxp3 expression and, instead, produce IL-17A and RANKL (20, 27); thus, making the inflammatory milieu highly enriched in inflammatory cytokines like IL-6, pivotal in dictating Treg lymphocyte fate in periodontal lesions.

The antagonistic relationship between bone-resorptive Th17 lymphocytes and immunoregulatory Treg lymphocytes dictates the delicate balance of alveolar bone remodeling (17, 20). On the one hand, osteoclasts differentiate and activate in the presence of macrophage colony-stimulating factor (M-CSF) and RANKL, which increase during periodontitis mainly by Th17 activity; while on the other hand, osteoblasts produce osteoprotegerin (OPG), the RANKL soluble decoy, which is partly mediated by Treg lymphocyte activity during periodontitis (17, 23, 27–31). Therefore, the cooperative action between bone-resorbing osteoclasts and bone-forming osteoblasts, regulated by the immune response, defines the dynamic maintenance of alveolar bone homeostasis.

## Current Animal Models for Experimental Periodontitis

The complexity and diversity of factors that contribute to periodontitis pathogenesis, including the combination of polymicrobial synergy and dysbiosis, chronic inflammatory dysregulation, and genetic predisposition factors have done the search for an ideal animal model of periodontitis difficult and challenging. Nevertheless, several authors have managed to mimic how these factors influence the development of periodontal diseases by separating them into different phases, including the formation of biofilms, bacterial colonization and invasion of periodontal tissues, induction of a deregulated host immune response, and breakdown of soft tooth-supporting tissues and alveolar bone resorption (32). **Table 1** summarizes the different methods that have been used to generate experimental periodontitis in mice. In general terms, the advantages of using mice models include a known microbiota and immune composition, diverse genetically engineered strains, and rapid availability of reagents for the investigation of recently described molecules (59). However, conditions such as substantially small mouths require highly skilled operators and a large number of animals (60).

**Table 1:** The most widely used models to generate experimental periodontitis in mice.

Experimental Periodontitis Model	Description	Requirements	Advantages and Biological Approaches		Limitations	References
			Technical Advantages	Biological Application and Plausibility		
Oral gavage/oral infection	Inoculation of live human bacteria, such as *Pg*, *Aa, and/or Fn*, via an oral-esophageal-gastric cannula or a micropipette into the mouse digestive system. • Gut microbiota dysbiosis and bacteremia favors chronic low-grade inflammation similar to periodontitis.	1) Anaerobic/capnophilic culture and bacteria-compatible animal facilities. 2) Constant monitoring and standarization of bacteria MOI. There is no consensus regarding the ideal concentration or quantity of inoculated bacteria.	1) Allows the precise enteric administration of bacteria. 2) It can be performed without anesthesia.*	1) Promotes periodontal inflammation and progressive alveolar bone resorption consistent with a chronic form of periodontitis. 2) Allows the bacterial invasion of mice oral tissues and bacteremia during a relatively long period of time (4-8 weeks). 3) The sustained systemic microbial challenge and low-grade systemic inflammation resembles the chronicity of periodontitis. 4) Enables the study of the association between systemic conditions and periodontitis-associated bacterial strains. Depending on the MOI and bacterial strain, it can also favor gut dysbiosis, joint inflammation, atheroma formation, and neuroinflammation.	1) Not fully effective to induce periodontal lesions. It generates less alveolar bone loss compared with other models. 2) Multiple inoculations in a long period of time (4-8 weeks) are needed until disease development. Increased animal stress and risk of esophageal lesions. 3) Effectiveness depends on the used bacterial strain and its virulence. 4) Not fully compatible with immunocompromised mice strains and humanized mice models.	(7, 25, 33–39)
Periodontal inoculation of bacteria	Localized microinjection of live human bacteria into mouse vestibular or palatal mucosa. • Mucosal infection and local bacterial challenge induce a local immune response capable of generating periodontal lesions.	1) Anaerobic/capnophilic culture and bacteria-compatible animal facilities. 2) Constant monitoring and standardization of bacteria MOI. 3) Constant anesthesia supplementation and post-intervention animal surveillance. There is no consensus regarding the ideal concentration or quantity of inoculated bacteria.	1) Semi-precise local administration of bacteria.	1) Promotes periodontal inflammation and alveolar bone resorption consistent with a chronic form of periodontitis. 2) Enables the study of specific periodontal host-bacteria interactions associated to infection, such as PRR-antigen interaction. 3) Useful for the study of virulence/immunogenic/pathogenic differences between periodontitis-associated bacteria.	1) Not fully effective to induce periodontal lesions. It generates less alveolar bone loss and inflammatory response compared with other models, such as ligature. 2) Repetitive injection regimen (2-3 per week) and mid-long experimental period until disease development (20-45 days).	(6, 18, 35, 40–42)
Oral and anal inoculation of periodontitis-associated bacteria	Topical administration of a mixture of 3%CMC and periodontitis-associated bacteria, such as *Pg*, into the mouse oral cavity and anus. • Mice coprophagia promotes continuous re-infection and establishment of chronic oral microbial challenge.	1) Anaerobic/capnophilic culture and bacteria-compatible animal facilities. 2) Constant monitoring and standarization of bacteria MOI.	1) Minimal or no trauma to the mouse mucosa. 2) It can be performed without anesthesia.*	1) Allows the bacterial invasion of mice oral tissues and bacteremia during a relatively long period of time (4-8 weeks). 2) Promotes periodontal inflammation and alveolar bone resorption consistent with a chronic form of periodontitis. 3) The sustained systemic microbial challenge and low-grade systemic inflammation resembles chronicity of periodontitis. 4) Enables the study of the association between systemic affections and periodontitis-associated bacterial strains.	1) Unprecise administration of bacteria. 2) Gut dysbiosis and faecal bacteria can be confounding factors. 3) Consecutive application regimen (8 days) and mid-long experimental period until disease development (8 weeks). 4) Not fully compatible with immunocompromised mice strains and humanized mice models.	(43–45)
Periodontal inoculation of isolated bacterial antigens	Local microinjection of known bacterial components, derived or not from periodontitis-associated bacteria, such as LPS. The inoculation is carried out into mouse vestibular or palatal mucosa. • Bacterial antigens directly elicit the mouse immune response.	1) Skilled operator. 2) Constant anesthesia supplementation and post-intervention animal surveillance. There is no consensus regarding the ideal concentration or quantity of inoculated bacterial antigen for the model.	1) It does not need bacterial culture^†^ or their inoculation. 2) Compatible with immunocompromised mice strains and humanized mice models.	1) Promotes periodontal inflammation and alveolar bone resorption consistent with an acute/aggresive form of periodontitis. 2) Induces low-grade systemic inflammation (in the case of LPS) when applied for at least 2 weeks. It can provoke cortical lesions, neuroinflammation, and arthritic lesions. 3) Enables the study of the specific interaction of PAMPs with the host immune response.	1) Repetitive injection regimen (2-3 per week). 2) It does not emulate bacteria-host interaction, essential during periodontitis.	(18, 46–50)
Chemically-induced periodontitis	TNBS and/or DSS are orally delivered weekly and/or biweekly. • DDS targets the innate immune response by undermining the epithelial barrier and inducing ROS production. TNBS induces a T-cell mediated response.	1) Experiment can last between 7 to 18 weeks, with weekly or biweekly interventions.	1) It does not need anesthesia nor bacterial culture. 2) Compatible with immunocompromised mice strains and humanized mice models.	1) Promotes periodontal inflammation and progressive alveolar bone resorption consistent with a chronic form of periodontitis. 2) Induces low-grade systemic inflammation, including colon and liver lesions. 3) Allows the study of the association between gut mucosal and oral mucosal inflammation.	1) It does not emulate bacteria-host interaction, essential during periodontitis. 2) Not fully effective to induce periodontal lesions. It generates less alveolar bone loss compared with other models, such as ligature.	(51, 52)
Ligature-induced periodontitis	Placement of a retentive ligature, usually silk, around or at the interproximal spaces of the mouse tooth. • Accumulation of bacterial biomass favors the development of a dysbiotic microbiota capable of inducing a local mucosal immune response and periodontal lesions.	1) Highly skilled operator, with optional magnification devices. 2) Most models use silk sutures around maxillary second molars, though there is no consensus regarding the place or width/length of the ligature or the need of its renewal.	1) Compatible with immunocompromised mice and humanized mice models. 2) Minimal trauma to the mouse mucosa. 3) Allows the collection of mouse gingival crevicular fluid.	1) Promotes acute periodontal inflammation and rapid alveolar bone resorption resembling an acute/aggresive form of periodontitis. 2) Compatible with the current oral dysbiosis-associated periodontitis pathogenesis paradigm. 3) Induces low-grade systemic inflammation, also compatible with periodontitis definition. 4) Allows the study of local immune response against inespecific bacterial challenge and alveolar bone regeneration after ligature removal. 5) When combined with oral gavage or periodontal inoculation, it can be useful for the study of virulence/immunogenic/pathogenic differences between periodontitis-associated bacteria.	1) Risk of mechanical trauma if not performed by calibrated operator. 2) Animals need to be constantly checked for ligature position. 3) No sustained bone loss after prolonged periods of time, unless combined with bacteria inoculation or gavage; thus, not resembling periodontitis chronicity.	(20, 23, 29, 32–35, 53, 54)
Calvaria inoculation of periodontitis-associated bacteria	Subcutaneous inoculation of periodontitis-associated bacteria, mostly *Pg*, at the skull midline between the ears and eyes. • The injection induces the formation of an abscess, acute local inflammation, and adjacent bone resorption.	1) Anaerobic/capnophilic culture and bacteria-compatible animal facilities. 2) Constant monitoring and standarization of bacteria MOI. There is no consensus regarding the ideal concentration or quantity of inoculated bacteria.	1) Semi-precise local administration of bacteria. 2) Does not require a skilled operator.	1) Promotes acute subcutaneous inflammation and rapid alveolar bone resorption, resembling an acute/aggresive form of infection/inflammation-induced bone resorption. 2) Allows the study of the immunogenic and pathogenic potential of bacteria.	1) Abscess formation does not resemble a periodontitis lesion. 2) Not fully compatible with immunocompromised mice strains and humanized mice models.	(55–58)

Aa, Aggregatibacter actinomycetemcomitans; CMC, carboxymethyl cellulose; DSS, dextrane sulfate sodium; Fn, Fusobacterium nucleatum; LPS, lipopolysaccharide; MOI, multiplicity of infection; PAMPs, pathogen-associated molecular patterns; Pg, Porphyromonas gingivalis; PRR, pattern recognition receptor; ROS, reactive oxygen species; TNBS, 2,4,6-Trinitrobenzene sulfonic acid.

*The use of isofluorane anesthesia is recommended in some publications to reduce the provoked stress and the incidence of esophageal lesions during oral gavage.

^†^Some authors would prefer to extract bacterial components, such as LPS, from their own bacteria cultures.

Experimental mice models developed for the study of periodontitis have been substantially beneficial and important to examine diverse biologic hypotheses related to disease pathogenesis, host-bacteria interactions, and therapeutic approaches, being able to, at least in part, finely reproduce the clinical, molecular, and histologic features of human periodontitis. Besides, the animals used to generate periodontitis are relatively inexpensive, easy to handle, have a short gestation period, and are characterized by developing a highly reproducible periodontal inflammatory process (33, 61). Among the various methods employed to mimic periodontitis in mice, currently, the most commonly used are oral gavage, periodontal inoculation, and ligature.

### Oral Gavage

The oral gavage model consists of the inoculation of human bacterial strains with an oral-esophageal gauge or a micropipette, usually using 10^9^ colony-forming units in a viscous suspension, prepared with 2% carboxymethylcellulose (62). The bacteria that have been most frequently used to induce periodontitis by oral gavage are *P. gingivalis*, *A. actinomycetemcomitans*, and *F. nucleatum*, as well as combinations of different bacterial strains (63–66). This method has been used successfully to establish the relationship between periodontitis and systemic conditions. For instance, the inoculation of *P. gingivalis* in hyperlipidemic mice *via* oral gavage provoked the accelerated formation of atherosclerotic plaques (67).

However, this experimental design can last at least 4 and up to 8 weeks until clear and significant evidence of alveolar bone resorption is achieved. Moreover, the magnitude of bone loss is not always reproducible, and the frequent inconsistency of the results is attributable to various factors, being the systemic nature of the infection caused the main factor (33, 34, 61). In consequence, oral gavage has been established as not fully effective to induce periodontitis (34).

### Periodontal Inoculation

The periodontal inoculation model comprises the localized microinjection of bacteria or some isolated bacterial component, such as lipopolysaccharide, directly into the palatal interproximal gingiva between the first, second, and third maxillary molar (18, 35). This method promotes significant periodontal inflammation, characterized by an increased expression of pro-inflammatory cytokines, apical migration of the junctional epithelium, and activation of osteoclastogenesis, consistently resulting in alveolar bone resorption (36, 40). Regarding the injection regimen, evidence commonly shows that the injections are performed two or three times per week under general anesthesia, generally using isoflurane (61, 68). The experimental period for this methodology may vary according to the purpose of the study, being generally between 20 and 30 days, although significant evidence of alveolar bone loss can be verified 7 days after initiation of the injections (18, 69).

This model has been used to evaluate different hypotheses regarding the mechanisms of periodontal inflammation and alveolar bone loss, due to the fact that it allows a reliable characterization of the immune response induced in the periodontal tissues and the cervical lymph nodes that drain the infected periodontium (6, 40). Since the mono-infection with a known bacterium allows great experimental control over the pathogenic stimulus, this model has been shown to be useful for analyzing pathogenic differences between different periodontal bacteria, their different serotypes, or bacteria defective in a certain virulence factor (6, 7, 18, 40, 41).

### Ligature

Ligature-induced periodontitis is an efficient model capable of inducing predictable alveolar bone loss within few days in mice (70). Besides, the removal of ligatures allows the study of lesion healing and resolution of inflammation (53). The main procedure involves placing silk, nylon, or cotton ligatures around maxillary or mandibular molars under anesthesia so that the retentive ligatures facilitate the accumulation of bacteria and thus, provoke periodontal inflammation and alveolar bone loss (53, 61, 71). Indeed, the occurrence of these bacteria-host interactions is compatible with the current paradigm of periodontitis pathogenesis, in which the development of dysbiotic oral microbiota provokes a bone-destructive immune response (28, 72).

The use of ligatures to induce periodontitis in mice has permitted the kinetic analysis of the morphologic characteristics of bone resorption (53). Differences between control and ligated mice alveolar bone loss become evident after just 5 days and gradually increase their differences at day 10 and 15 (20). Apart from that, the characterization of bacterial accumulation in ligatures has revealed that mice oral commensal communities go through extensive structural and composition changes, leading to microbial dysbiosis (28). Meanwhile, differential immune responses can be appreciated at subsequent time points; for instance, myeloperoxidase-producing cells, such as neutrophils, can be seen since day 3, and genes related to the critical adaptive immune response in periodontitis have expression peaks in periodontal lesions after 9 days, including markers for Th1, Th2, Th17, and Treg lymphocyte activity (32).

The ligature-induced periodontitis model has been extensively reported in mice (20, 23, 29, 32–35, 53, 54, 60); however, the extremely small mice oral cavity and the minuscule interproximal space between the maxillary or mandibular molars represent a not minor technical difficulty in placing the ligatures (73). Moreover, this placement can get even harder when dealing with immunodeficient mice when working in sterile conditions; thus, reinforcing the idea that experienced and highly skilled operators are needed. In response to this, simpler ligature models have been proposed, including the use of a 0.2-mm orthodontic ligature wire, after filing the molar interproximal surfaces with a curved C+ nickel-titanium root canal file, and the use of already tied ligature knots and 3D-printed devices (32, 74). These preformed ligatures would allow their atraumatic positioning in any molar interdental space, accumulating plaque and provoking a local immune response followed by bone resorption, in a similar way to its predecessors (32).

## Translational Limitations of the Current Animal Models for Experimental Periodontitis

Even though the above-described animal models have proven to be particularly useful to study the molecular mechanisms associated with the onset, progression, and recovery from periodontitis, different challenges also arise when we try to translate these results to human contexts. Despite the similarities between mice and human immune systems, including molecular mediators and cell subpopulations that interact during both innate and adaptive immune responses (59), there are still discrepancies that must be considered. Indeed, substantial molecular differences between murine and human growth factors and cytokines influence the course of their hematopoietic and immune system development (75). Among the most relevant differences, gingival microbiota, key molecular mediators, including immunoglobulins, cytokines, chemokines, co-stimulatory molecules, Toll-like receptors, and T-cell signaling pathways should be considered when a translational projection of results obtained in animal models of periodontitis is desired (28, 75, 76). For instance, human effector CD4^+^ T lymphocytes express human leukocyte antigen (HLA) molecules and regulate Foxp3 expression without necessarily acquiring an immunoregulatory phenotype, while mouse Foxp3^+^ T lymphocytes are mostly defined as regulatory (75, 76); thus, displaying variations that could be important in the study of periodontitis. Furthermore, the ethical and technical constraints that severely limit *in vivo* studies of human biology, constant evolution of human-specific diseases, resistance to microbial infections, and pharmacodynamic interactions have led to increasing demand for translationally enhanced animal models. In this context, the limitations that hinder the direct replication of outcomes predicted by murine studies into human diseases support the requirement for the generation of humanized mouse models for the study of periodontitis (77).

## Humanized Mice Models

**Figure 1:**
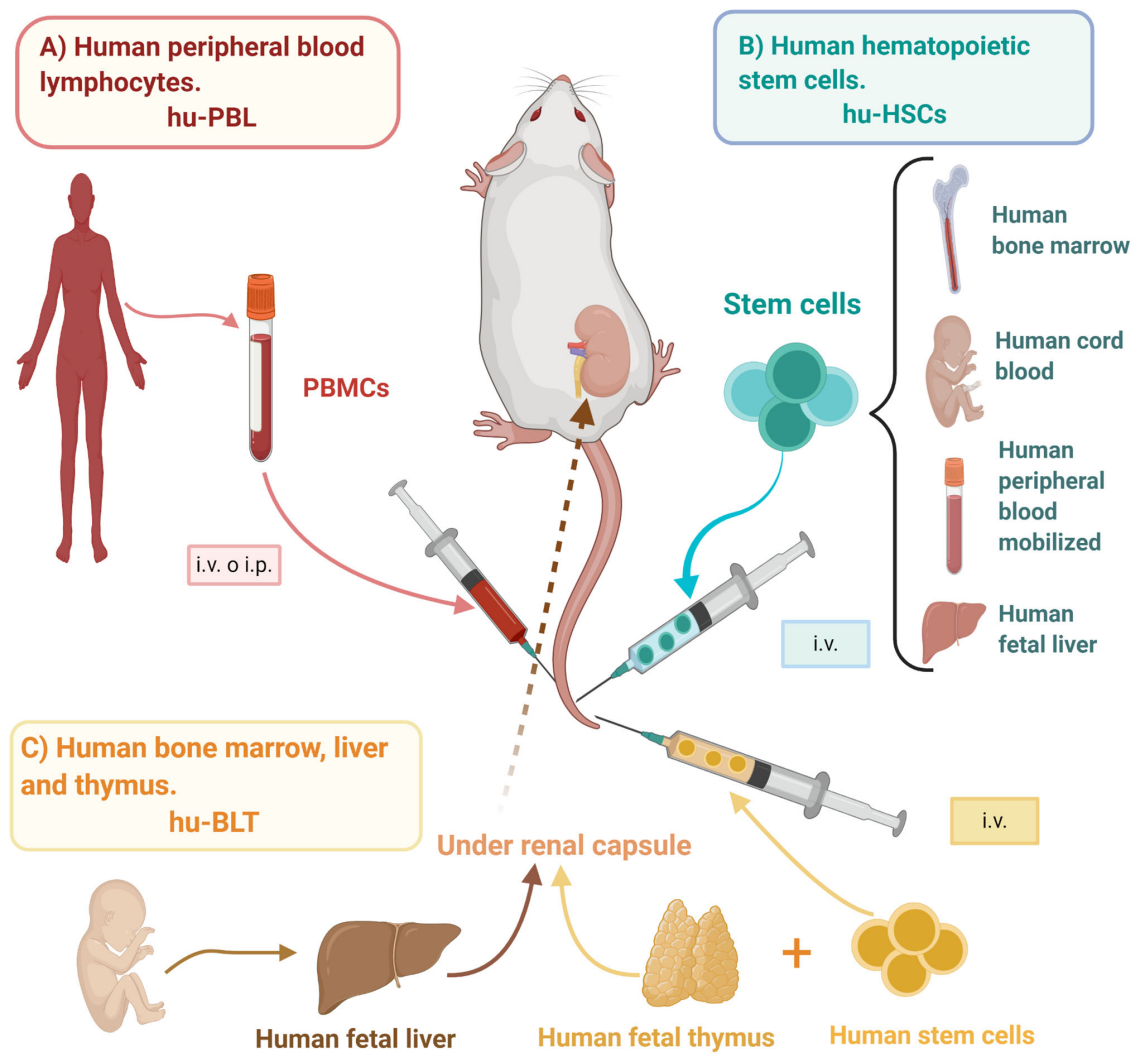
Mice humanization strategies. Mice/human chimeras originate from immunodeficient mice engrafted with different sources of human cells or tissues able to reconstitute a human-like immune response, such as **(A)** hu-PBL: PBMCs are obtained from healthy donors and i.v. or i.p. inoculated, **(B)** hu-HSCs: HSCs may be obtained from bone marrow, umbilical cord blood, peripheral blood, or fetal liver, and i.v. inoculated into either adult or newborn mice, and **(C)** hu-BLT: Bone marrow stem cells and tissues from fetal liver and thymus are transplanted into previously irradiated mice, specifically under the renal capsule. hu-BLT, human bone marrow, liver, and thymus; hu-HSCs, human hematopoietic stem cells; hu-PBL, human peripheral blood lymphocytes; i.p., intraperitoneal; i.v., intravenous; PBMCs, peripheral blood mononuclear cells. Created with BioRender.com.

Humanized mice models, or mouse/human chimeras, have been defined as immuno-deficient mice engrafted with human primary hematopoietic cells and/or tissues capable of reconstituting a functional human immune system (HIS) (75); thus, providing an opportunity for the study of live human biological processes and affections. There are three main humanized mice models: the human peripheral blood lymphocytes (hu-PBL) model, the human stem cells (hu-HSC) model, and the human bone marrow/fetal liver/thymus (BLT) model (**Figure 1**), each with its distinctive advantages and limitations (**Table 2**).

**Table 2:** The most widely used models for mice humanization.

Humanized Mice Model	Description	Requirements	Advantages and Biological Approaches		Limitations	References of its use in periodontitis studies
			Advantages	Biological Application and Plausibility		
Human peripheral blood lymphocytes (hu-PBL) model.	Inoculation and engraftment of PBMCs, via intravenous, intraperitoneal, intrafemoral, intracardiac, or intrahepatic injection.	Preconditioning with a sublethal dose of irradiation facilitates human cell engraftment (Optional).	1) The easiest and most cost-efficient method for mice humanization. 2) Abundance of human PBMCs available for mice engraftment. 3) Fast human cell engraftment kinetics. Human cells are observed in mice blood within days and up to 4 to 6 weeks after their inoculation.	1) Effector and memory T lymphocytes are the main human cell populations present in this model. Method of choice for the analysis of CD3^+^ T lymphocytes. • T lymphocytes, particularly Th17 lymphocytes, have a vital role during oral mucosal immune surveillance and periodontitis immune response, by producing IL-17A, chemoattracting neutrophils, and promoting RANKL upregulation.	1) Short experimental window, due to rapid onset of GvHD (4 to 8 weeks). 2) GvHD is faster if preconditioning irradiation is performed. 3) Low engraftment of primary immune response cells.	(78–84)
Human stem cells (hu-HSC) model.	Inoculation and engraftment of CD34^+^ HSCs obtained from bone marrow, cord blood, or fetal liver, via intravenous, intrafemoral, intracardiac, or intrahepatic injection.	Preconditioning with a sublethal dose of irradiation allows the depletion of mouse HSCs and facilitates human HSCs engraftment (Conditional to mouse strain).	1) Allows the humanization of adult and newborn mice. 2) Mouse strains with mutations in receptor c-Kit or transgenic expression of SCF allow the successful engraftment of human HSCs without the necessity of preconditioning irradiation.	1) Allows the engraftment of human erythrocytes, platelets, T lymphocytes, NK cells, dendritic cells, monocytes/macrophages, and granulocytes. • The granulocyte (neutrophil)/Th17 lymphocyte axis is vital during oral mucosal immune surveillance and periodontitis immune response. • Monocyte/macrophage subpopulations, including subsets M1 and M2, have a role in pro-inflammatory cytokine production and inflammation resolution/healing during periodontitis. • Dendritic cells are the major antigen-presenting cells during periodontitis. • NK cells have a role during periodontal inflammation.	1) Limited engraftment of B lymphocytes, and if it occurs, they are generally non-functional. 2) Impaired immune cell differentiation due to lack of thymic HLA.	No study.
Human bone marrow/fetal liver/thymus (BLT) model.	Surgical transplantation of human fetal liver and thymus fragments under the kidney capsule of mice, followed by an intravenous injection of human HSCs.	Preconditioning with a sublethal dose of irradiation allows the depletion of mouse HSCs and facilitates human HSCs engraftment (Necessary).	1) Development of a robust mucosal human immune system. 2) Promotes an enhanced reconstitution of secondary lymphoid organs. 3) Reconstitution of lymph nodes allows the constant repopulation of human immune cells.	1) Allows the engraftment of human T lymphocytes, B lymphocytes, monocytes, macrophages, and dendritic cells. 2) Very useful for the study of human T lymphocytes, due to the fact that these cells maturate in the transplanted autologous thymic tissues. • The development of a robust human-like mucosal immune system could be compatible to emulate an intricate network of human-like periodontal immune responses, with a constant expansion and activation of resident and infiltrating immune cells, similar to human periodontitis lesions.	1) High incidence of GvHD, that limits the time window for experimentation.	No study.

GvHD, xenogeneic graft-versus-host-disease; HLA, human leukocyte antigen; PBMCs, peripheral blood mononuclear cells; SCF, stem cell factor; NK, natural killer.

### Human Peripheral Blood Lymphocytes (hu-PBL) Model

The hu-PBL model consists of the inoculation and engraftment of human leukocytes isolated from peripheral blood, also termed peripheral blood mononuclear cells (PBMCs), *via* intravenous (i.v.), intraperitoneal (i.p.), intrafemoral (i.f.), intracardiac (i.c.), or intrahepatic (i.h.) injection. This is the easiest and most cost-efficient method of animal humanization due to the large quantities of human leukocytes that can be isolated from peripheral blood. Also, this model has fast engraftment kinetics, as human leukocytes can be found circulating in murine peripheral blood within days and up to 4 to 6 weeks (75, 76). Human T lymphocytes with an activated effector and memory phenotype are the main population present in this model, whereas B lymphocytes and myeloid cells are present but at much lower quantities, probably due to the dominant expansion of T lymphocytes and the lack of human cytokines required for their survival (76, 85, 86).

Technically, mice may be preconditioned with a sublethal dose of irradiation, which has been reported to facilitate engraftment and colonization of human PBMCs. Nonetheless, this step is not completely necessary for all mice platforms because the PBMC inoculum contains already mature human leukocytes that do not need to undergo differentiation in the mouse environment. Moreover, irradiation accelerates the occurrence of xenogeneic graft-*versus*-host-disease (GvHD), which results in reduced animal survival (87). In this context, rapid onset of GvHD is the main disadvantage of the hu-PBL model, generated by the elevated levels of activated human T lymphocytes due to MHC I and II mismatch. However, new strains of mice deficient in MHC I and/or II delay GvHD development and increase animal survival, thus widening the available experimental window (88).

### Human Stem Cells (hu-HSC) Model

In the hu-HSC model, human hematopoietic stem cells (HSC) are injected and engrafted into either adult or newborn immunodeficient mice (89, 90). The CD34^+^ HSCs may be obtained from bone marrow, cord blood, fetal liver, or mobilized human HSCs and are injected i.v. or i.f. into adult mice or i.v. (facial vein), i.c., or i.h. into newborn mice (91, 92). Additionally, for this model to achieve effective levels of HSC engraftment, myelosuppression preconditioning with sublethal irradiation is necessary to deplete mouse HSCs. However, new mouse strains with mutations in c-Kit, a stem cell factor (SCF) receptor critical for HSC engraftment, or with transgenic expression of membrane-bound human SCF allow the successful engraftment of human HSCs without previous irradiation (93, 94). After preconditioning and inoculation, a diverse repertoire of cell populations differentiates into multiple lineages of human cells and engrafts the murine tissues. Those cells include erythrocytes, platelets, T lymphocytes, natural killer (NK) cells, dendritic cells, monocytes/macrophages, and granulocytes (89, 90, 95–97). Nevertheless, this model presents some important disadvantages, such as the lack of a functional B lymphocyte compartment, in part due to inadequate CD4^+^ T lymphocyte function and impaired antigen response. This is associated with the lack of HLA on the thymic epithelium and the absence of human primary lymphoid organs, and consequently, the limited differentiation of human cells inside the model.

### Human Bone Marrow, Liver, and Thymus (hu-BLT) Model

The hu-BLT model consists of the surgical transplantation of human fetal liver and thymus fragments under the kidney capsule of sublethally irradiated immunocompromised mice, followed by an i.v. injection of autologous HSCs (98). This model has been described as superior to the others, as it promotes an enhanced reconstitution of secondary lymphoid organs, which contributes to HIS education and allows the systemic repopulation of multiple human immune cell lineages, including T and B lymphocytes, monocytes, macrophages, and dendritic cells. This model has been an important tool to study human T lymphocyte development, as these cells are educated in autologous thymic tissues (99, 100). Nevertheless, the hu-BLT model presents higher GvHD incidence compared with the hu-SRC model, sometimes earlier than 20 weeks after transplantation (101).

## Immunodeficient Murine Hosts as Platforms for Humanization

Besides the humanization strategy, successful engraftment largely relies on the features of the animal recipient host. In this context, the development of immunodeficient mice hosts, capable of engrafting human cells or tissues, has implied a progressive succession of genetic modifications in order to avoid xenogeneic graft rejection and enable a stable reconstitution of human cells (76, 102). In brief, one of the first groundbreaking achievements was the *Prkdc^scid^* (protein kinase, DNA activated, catalytic polypeptide) mutation on the CB17 mouse strain, commonly named as SCID (severe combined immunodeficiency) (103). This mutation results in a reduced number of functional T and B lymphocytes, enabling transient engraftment of human PBMCs, HSCs, or fetal hematopoietic tissues (92, 104). Nevertheless, with aging, these mice generate autologous T and B lymphocytes, an event termed leakiness. Moreover, this animal host still presents high levels of NK cells and other innate immune cells that hinder proper human cell engraftment (76, 102).

Another approach implied the targeted mutation of the recombination-activating genes 1 and 2 (*Rag1* and *Rag2*), which impedes the development of functional T and B lymphocytes in mice and prevents leakiness (105, 106). However, Rag1/2-deficient mice maintain a high NK cell activity, allowing limited engraftment of HSCs (76). Afterward, NOD-SCID mice were developed by crossing NOD (for non-obese diabetic) mice with the SCID strains (107). This animal host presents additional defects in innate immunity, such as the lack of complement C5, and impaired macrophage cytokine production, antigen presentation, and NK function (76, 107, 108). Even though this approach provides enhanced human HSC and PBMC engraftments (109, 110), the model also has a limited life span due to the early development of lymphomas and innate immunity residual activity mice strain (76).

### NSG, NOG, and BRG Mice

**Figure 2:**
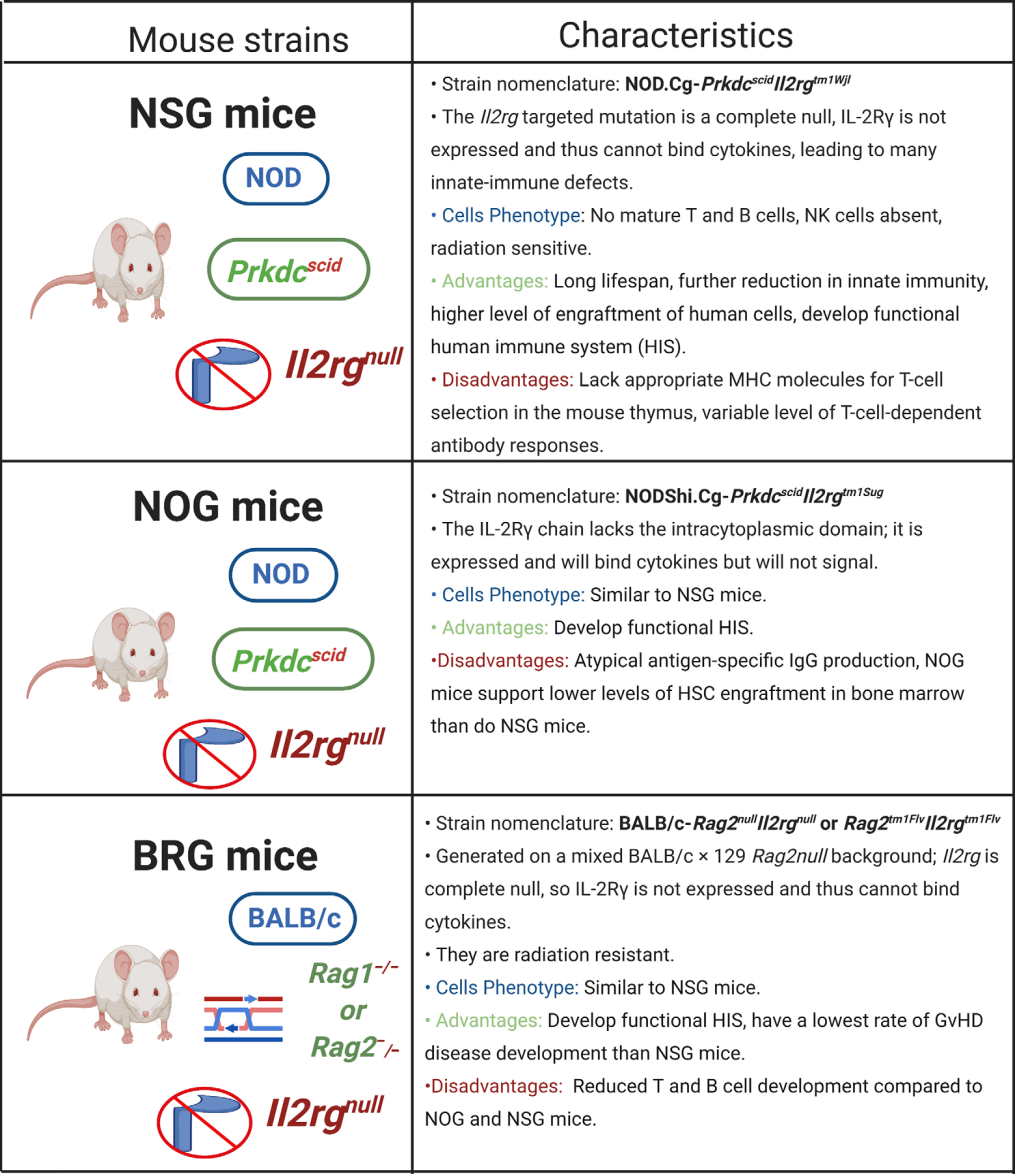
Immunodeficient mice strains prone to humanization. Several genetic modifications have enabled that immunodeficient host mice be capable of engrafting human cells or tissues without immediate xenogeneic rejection and allow a stable reconstitution of human cells. These immunodeficient mice include NSG mice, NOG mice, and BRG mice, each one with its own advantages and disadvantages summarized in the Figure. *il2rg*, interleukin-2 receptor subunit gamma; MHC, major histocompatibility complex; NOD, non-obese diabetic. Created with BioRender.com.

Fortunately, another immunodeficient mouse strain with a targeted mutation of the *Il2rg* gene, which encodes IL-2 receptor γ-chain (IL-2Rγ), has been developed (89, 90, 95, 111, 112) (**Figure 2**). IL-2Rγ is an essential component for IL-2, IL-4, IL-7, IL-9, IL-15, and IL-21 signaling and its absence in *Il2rg^null^* mice leads to defective lymph nodes, deficient T and B lymphocyte development, affects innate immunity, and completely abolishes NK cell generation (113, 114). Besides, this mutation has promoted enhanced support of both human HSC and PBMC engraftments as compared with the previously described immunodeficient mice (76). After an extensive succession of mutations, the main immunodeficient mouse strains currently used are NOD-SCID *Il2rg^null^* mice, which includes NOD.Cg-*Prkdc^scid^Il2rg^tm1Wjl^* (NSG mice), NODShi.Cg-*Prkdc^scid^Il2rg^tm1Sug^* (NOG mice), NOD-*Rag1^null^ Il2rg^null^* (NRG), and BALB/c-*Rag2^null^Il2rg^null^* or *Rag2^tm1Flv^Il2rg^tm1Flv^* (BRG mice). All these mice strains are able to support human tissue and cell engraftment (86, 90, 95). The characteristics, advantages, and disadvantages of each immunodeficient mouse model derived from *Il2rg^null^* mice have been extensively reviewed in detail by Shultz et al. (75, 76).

Altogether, these animal models comprise an important opportunity for the study of multiple biological processes and diseases, including periodontitis. Importantly, the selection of a model or another depends on the scientific question and experimental settings; thus, it is highly recommended to consider that the levels of functional HIS engraftment differs depending on the recipient mouse strain and humanization strategy used.

## Current Challenges for Mice Humanization

Notwithstanding the accumulated successes achieved along with the development of new and more refined immunodeficient murine platforms, the development of a vigorous functional HIS following engraftment, necessary for transferable humanized models, has remained challenging. This is due to species specificity of MHC antigens, homing molecules that may impede appropriate trafficking of human immune cells, discrepancies between hematopoietic growth factors and cytokines, poor development of lymphoid architecture, and impaired class switching and affinity maturation of immunoglobulins (115).

In this context, mice humanization protocols that involve the transference of human cells into severely immunodeficient mice strains may initiate an alloreactive response in which T lymphocytes react against HLA disparities and minor antigens, resulting in the initiation of GvHD. This potentially life-threatening complication consists in the acute anti-host effector response of human T lymphocytes recognizing foreign murine MHC expressed by host recipient cells (88). While the rapid development of this disease enables preclinical testing of human immunosuppressive agents, the relatively short survival of engrafted animals given by GvHD could prevent the realization of long-term *in vivo* studies and the proper emulation of chronic diseases (101, 115).

In other words, as mature CD4^+^ T lymphocytes have been educated in the human thymic stroma, they are not tolerized to the murine antigenic environment, which leads to the rapid-onset of xenogeneic GvHD. In fact, during engraftment of human cells, GvHD onset and severity vary between donors, which seem to depend on the number of CD4^+^ T lymphocytes within the transferred human cells (116). Another important influencing factor is the mice strain, from which NSG mice provide a faster expansion of the human CD45^+^ compartment and higher engraftment levels of CD3^+^ T lymphocytes; however, they also have a faster rate of GvHD than, for example, BRG mice (117). Also, HSC-reconstituted mice might not be able to recognize antigens presented by HLA-DR human dendritic cells in the periphery because they are specific for murine MHC class II molecules. This negatively affects the induction of an efficient immune response, resulting in reduced Th lymphocyte activity and insufficient interactions between T and B lymphocytes, which are required for class-switch recombination (116). Thus, substantial considerations should be taken into account when selecting the ideal model to assess the immunopathogenesis and the efficacy of novel immunotherapies for periodontitis.

Despite these limitations, a significant improvement has been accomplished by the transgenic introduction of human HLA molecules into immunodeficient mice strains. For example, transgenic expression of HLA-DR4 in NRG mice has enabled the proper development of CD4^+^ T lymphocytes and completely functional B lymphocytes from infused HSCs of HLA-DR-matched donors (118). In addition, the complimentary removal of murine MHC class II molecules, the main target of human CD4^+^ T lymphocyte-mediated GvHD responses, further improved the generation of human antigen-specific immune responses in immunodeficient mice reconstituted with human cells while reducing the risk of xenogeneic GvHD development (119). Therefore, the diversity of humanized model systems represents an important set of tools for modeling pathogen interactions with human cells and tissues *in vivo* (120), so the consecutive overcome of their punctual limitations enlightens their potential application for the study of immunopathologies characterized by T lymphocyte-mediated aberrant responses, such as periodontitis.

## Characterization of the Humanized Mice Models

Following the transplantation of human cells, an extensive engraftment characterization is often required. Indeed, clinical features of the recipient host, donor cell source, and engraftment technique have a great influence on the kinetics, extent, composition, and morphological aspects of the graft reconstitution in the different organs and, consequently, its consistency. For instance, NSG mice exhibit small, poorly developed primary and secondary lymphoid organs, which lack typical lymphoid structures and are solely composed of reticular stromal cells (75, 121). Consequently, grafted human cells, which form variably sized aggregates, are not capable of recreating the typical lymphoid tissue architecture of immunocompetent organisms (75, 121, 122). Therefore, extensive immune profiling across innate and adaptive immune cell subpopulations, including human T lymphocytes (CD3^+^, CD4^+^, and CD8^+^ cells), B lymphocytes (CD19^+^ cells), macrophages (CD68^+^ cells), and neutrophils (CD15^+^ cells), among others, should be characterized before studying the development periodontitis or any proposed therapeutic approach (123, 124).

**Figure 3:**
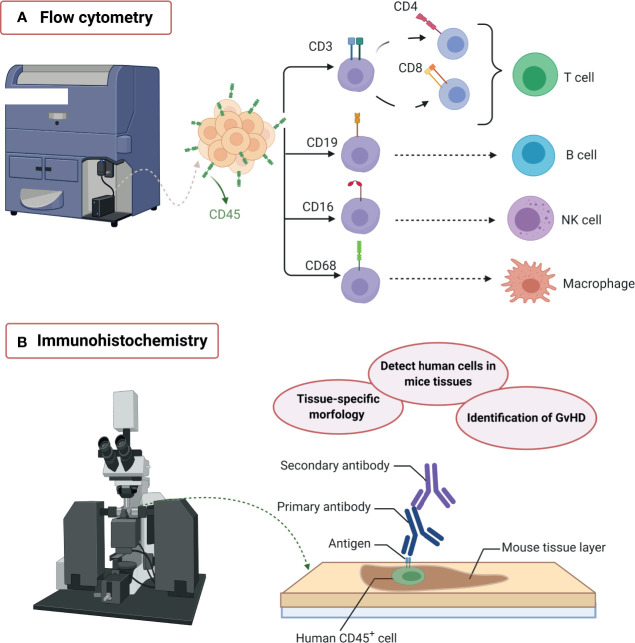
Humanized mice model characterization. The diversity of graft sources and receptors for humanization often needs an extensive immune cell subpopulations’ profiling in order to discriminate the desired human-like immune response from the graft-*versus*-host-disease (GvHD) xenogeneic immune response. For this purpose, flow cytometry and immunohistochemistry have been used. **(A)** Flow cytometry: The use of multiple fluorochrome-labeled antibodies allows the identification of different immune cell lineages at the same time. The CD45^+^ peripheral blood mononuclear cells (PBMCs) can be further classified, for example, into CD3^+^CD4^+^/CD8^+^ T cells, CD19^+^ B cells, CD16^+^ natural killer cells, or CD68^+^ macrophages, which are characteristic of a human-like periodontal immune response. **(B)** Immunohistochemistry: Tissue labeling allows the spatial identification of human cells in mice tissues, the visualization of their interaction with periodontal tissue-specific morphology, and the identification of GvHD lesions. Created with BioRender.com.

To analyze human cell engraftment in immunosuppressed mice, flow cytometry is a cost-effective method for the characterization of cell populations through the identification of lineage-specific markers using fluorochrome-coupled antibodies (**Figure 3A**). Indeed, flow cytometry allows the analysis of isolated cells from different tissues or organs by quantifying the percentages and the absolute number of murine and human immune cells on each sample, starting with CD45^+^ cytometric gating strategy for immune cells subpopulations. Nowadays, flow cytometry technology has advanced to the capacity of measuring up to 50 parameters on a single cell; however, most flow cytometers are limited to 12-18 parameters per sample, and spectral overlap makes its analysis complex. Apart from that, immunohistochemistry analysis contributes with the tissue-specific spatial context to better understand engrafted human cell homing and distribution in mice tissues (**Figure 3B**), additionally helping to identify the GvHD inflammatory lesions dominated by T lymphocytes and macrophages (125–127).

Despite the important information that flow cytometry and immunohistochemistry provide for the characterization of humanized mice, new high-dimensional parameter analysis tools have been introduced to accurately describe and understand the complexity of these models (128). For instance, mass cytometry offers a single-cell analysis that couples flow cytometry with mass spectrometry and is able to evaluate up to 50 simultaneous parameters without spectral overlap using markers coupled with metal isotopes (129, 130). Another novel tool is imaging mass cytometry, which also uses metal-tagged antibodies, enhancing the imaging of up to 37 protein markers on fresh-frozen or formalin-fixed paraffin-embedded tissue sections (131). These and other complemental high throughput platforms could allow the performance of a multidimensional data analysis that could allow the identification of subtle changes in periodontal immune populations (132).

## Humanized Mice Models: Challenges for Their Application and Opportunities for the Study of Periodontitis

In the periodontal disease research arena, the use of humanized mice models has been increasingly gaining interest for their potential translational applicability. The first proposed model demonstrated that the engraftment of immunodeficient mice with hu-PBLs provided a conceivable animal model to study human immune responses towards periodontitis-associated antigens/pathogens (78–83).

Indeed, to characterize the human leukocyte response against the periodontopathogen *A. actinomycetemcomitans*, formerly termed *Actinobacillus actinomycetemcomitans*, a humanized animal model named *Aa*-hu-PBL-NOD/SCID was designed (83). NOD-SCID mice were reconstituted with periodontitis-affected patients-derived hu-PBLs and then orally inoculated with *A. actinomycetemcomitans*. From this experiment, relevant findings were obtained, including: i) The achievement of significant engraftment, between 30-60%, of human leukocytes (80); ii) After bacteria challenge, an increase in the number of B lymphocytes, CD4^+^ and CD8^+^ T lymphocytes, and monocytes/macrophages was observed in the periodontal compartment; however, without bacteria inoculation, human leukocytes were almost exclusively observed around bone marrow blood vessels in the proximity to the periodontium (80); iii) When CD4^+^ T lymphocytes were isolated from the mice oral mucosa, they revealed significantly higher activation and proliferation levels in response to bacterial infection, with a phenotype distribution similar to that observed in T-cells isolated from the periodontitis-affected donors (79); iv) An important *A. actinomycetemcomitans*-specific IgG antibody response was achieved and maintained over a 6 to 8 week period after bacteria inoculation (78); and v) A significant increment in the RANKL expression and alveolar bone resorption, as well as decreased OPG expression, was detected in response to *A. actinomycetemcomitans* infection, as compared with the absence of bacteria inoculation (82). Interestingly, when OPG was administered as a therapeutic strategy to protect mice against periodontitis, the levels of alveolar bone resorption were significantly reduced even after bacterial infection (78).

Afterward, Zhang et al. (83) assessed the role of suppressor of cytokine signaling (SOCS) molecules in *A. actinomycetemcomitans*-induced osteoclastogenesis by using a humanized model in which NOD/SCID mice were engrafted with hu-PBLs derived from periodontitis patients or age-matched healthy subjects. They achieved engraftment of ~30% of Hu-PBLs and concluded that the RANKL-mediated dendritic cell-related osteoclastogenesis was associated with an upregulation of SOCS and downregulation of SOCS3, the dominant-negative form of SOCS (83). Moreover, by using an HLA-DR1 humanized C57BL/6 mice model of periodontitis infected with *P. gingivalis*, the development of rheumatoid arthritis and its impact on bone density and systemic cytokine production were also analyzed (133). *P. gingivalis* gingival infection promoted a transient increase in the number of Th17 lymphocytes and higher systemic cytokine activity, femoral bone density loss, and production of anti-citrullinated protein antibodies, as compared with sham-infected mice controls (133). On the other hand, when human monocyte-derived dendritic cells were reconstituted in NSG mice to induce humanization, the exposure to a fimbriae-expressing mutant strain of *P. gingivalis* led to the formation of anti-apoptotic dendritic cells, which drove dampened Th1/Th17 responses and promoted a potent indoleamine-2,3-dioxygenase-dependent Treg response (84). In this context, the use of the humanized mice model accompanied by the *in vivo* tracking of pathogen-loaded dendritic cells contributed to validate the *P. gingivalis*-induced immunomodulation of dendritic cells and the intracellular bacteria persistence in distant organs. Particularly, *P. gingivalis*-primed dendritic cells promoted Treg activity to evade effector immune responses, by preventing the apoptosis of carrier dendritic cells and thus, favoring its systemic dissemination and survival (84).

Despite the huge progress achieved on revealing the pathogenesis of periodontitis using rodent models, the precise roles of human immune cells and molecular mediators have not been fully elucidated yet. Wild-type animal models have been extensively used to study the nature of immune response against periodontopathogens; nevertheless, oral mucosa colonizers in mice are not the same microorganisms found in human periodontal tissues (28, 134). In this context, human periodontopathogens inoculated in wild-type rodents or the induction of bacterial dysbiosis by the ligature placement around mice molars may not exactly reflect the host-microbiota interactions described in humans; thus, making the extrapolation of the findings to the human framework difficult and limited. Taking these antecedents into account, humanized mice models could provide robust physiological systems available to be exploited in the periodontal arena.

Indeed, the intricate inflammatory nature of periodontitis opens the possibility to propose the use of humanized mice models to specifically address scientific questions that might not be fully explained with conventional mice models. For instance, the first stages of the inflammatory response against periodontopathogens are enriched in mediators belonging to the innate arm of the immune response, which could be evaluated using the hu-HSCs model, as it allows the engraftment of hematopoietic precursors and further differentiation of a vast repertoire of immune cell populations (89, 90, 95–97). Nevertheless, if antigen presentation or dendritic cells are a matter of interest, this model may be inadequate as it lacks the HLA expression necessary for cell differentiation. Conversely, hu-PBLs mice models comprise a graft in which an important percentage of immune cells have already gone through a differentiation process (75, 76). Particularly, CD4^+^ T lymphocytes, the main cell population that settles into host tissues with this strategy, have an activated effector or memory phenotype, which means that they do not need to undergo cell differentiation into the mouse environment. In the case of periodontitis, CD4^+^ T lymphocytes have been identified as key cells that mediate the alveolar bone resorption (16–18); therefore, the hu-PBLs mice model would be useful to analyze committed immune-phenotype cells, not so for primary immune responses. In all these models, it is noteworthy that xenogeneic mismatch should be considered. Although it has been described from the first assays that an antigen-specific human immune response (both humoral and cell-mediated) can be achieved in SCID or NOD/SCID systems even in the presence of ongoing GvHD (135–137), the non-specific activation of human immune cells, detected, for example, as an IL-2 background production, could also occur (80). For this, MHC knockout and/or HLA transgenic mice platforms could represent a great opportunity to prevent or diminish any confounding factors given by GvHD development.

**Figure 4:**
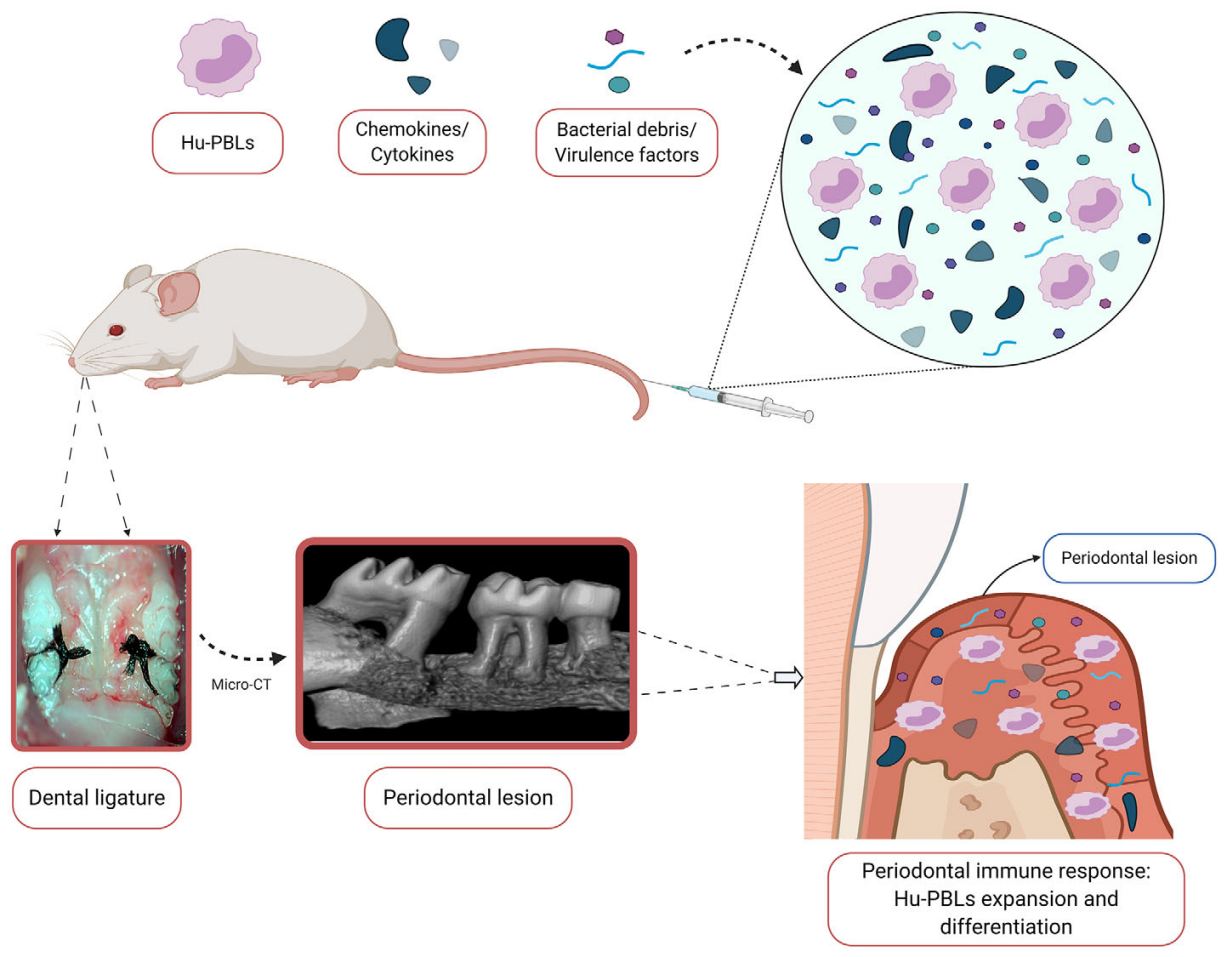
Proposal for a humanized mouse model for the study of periodontitis. The placement of bilateral silk ligatures around the second maxillary molars of hu-PBL-NSG mice provokes local bacteria accumulation, periodontal microbiota dysbiosis, and bacteremia. Consequently, this bacterial insult results in systemic dissemination of antigens, bacterial debris, virulence factors, cytokines, and chemokines. In turn, they are capable of being recognized by the engrafted human peripheral blood mononuclear cells (PBMCs), which ultimately leads to the orchestration of a human-like immune response within the periodontal tissues and lymph nodes that drain them. The resulting local expansion and differentiation of the human PBMCs would lead to the formation of a periodontal lesion, characterized by the presence of a dense inflammatory infiltrate and alveolar bone resorption. Hu-PBLs, human peripheral blood lymphocytes. Created with BioRender.com.

On the basis of the studies mentioned above, the use of humanized mice models for the study of the virulence of periodontal bacteria and the immunopathogenesis of periodontitis has proven to be feasible and particularly relevant (**Figure 4**). To ensure the success of this experimental strategy, it is highly recommended to adequately select both the murine platform and the humanization strategy, taking into account their advantages and limitations in the context of the study aims. In addition, a detailed characterization of the graft is necessary prior to any other experimental intervention, in order to ensure the standardization of humanization kinetics, adequate experimental work window, and reproducibility of the immunological findings developed in the host animal (115). Regarding therapeutic approaches, humanized animal models are an emerging topic in the field of periodontal research, so their development could be an important step in the search for innovative immunotherapeutic strategies applicable to periodontitis-affected patients.

## Concluding Remarks

The enhanced translational application and diversity of humanized mice models could innovate the way we study periodontal immune responses and consequently, prompt the designing of novel immunotherapeutics. However, there is an evolving need to overcome the complications related to the xenogeneic transfer of cells to immunodeficient hosts, which could compromise the study of this chronic disease and drug dynamics. Therefore, the proper characterization of the periodontitis humanized mice model should entail a high throughput analysis of the grafted human immune cells that repopulate the mice periodontium, potential antigen-presenting sites, and the remaining local and distal lymphoid organs. In this way, they could ensure staged profiling of the periodontitis-like immune response and avoid to the minimum its confusion with the GvHD-provoked immune response.

## Author Contributions

CR, EAC, and RV conceived the review. CR, EAC, and AP were involved in drafting the manuscript. MG designed and prepared the figures. LG-O, AS-C, and SM-R critically evaluated and supplemented the manuscript. EAC and RV revised and prepared the manuscript for submission. All authors contributed to the article and approved the submitted version.

## Funding

This study was financially supported by grant FONDECYT 1181780 (RV) from the Agencia Nacional de Investigación y Desarrollo (ANID), Chile. EAC, AS-C, and SM-R were the recipients of Ph.D. scholarships from the Faculty of Dentistry, Universidad de Chile, Chile. CR and LG-O were the recipients of Ph.D. scholarships Fondecyt 21180841 and 21190087, respectively, from ANID.

## Conflict of Interest

The authors declare that the research was conducted in the absence of any commercial or financial relationships that could be construed as a potential conflict of interest.
